# Effects of Daily Probiotics Supplementation on Anxiety Induced Physiological Parameters among Competitive Football Players

**DOI:** 10.3390/nu12071920

**Published:** 2020-06-29

**Authors:** A.M.G.C.P. Adikari, Mahenderan Appukutty, Garry Kuan

**Affiliations:** 1 Sports Science Programme, Faculty of Sports Science and Recreation, Universiti Teknologi MARA, Shah Alam, Selangor 40450, Malaysia; chandima@sjp.ac.lk (A.M.G.C.P.A.); mahen@uitm.edu.my (M.A.); 2 Department of Sports Science, Faculty of Applied Sciences, University of Sri Jayewardenepura, Nugegoda 10250, Sri Lanka; 3 Jeffrey Cheah School of Medicine and Health Sciences, Monash University Malaysia, Sunway, Selangor 47500, Malaysia; 4 Exercise and Sport Science Programme, School of Health Sciences, Universiti Sains Malaysia, Kubang Kerian Kelantan 16150, Malaysia; 5 Department of Life Sciences, Brunel University, London UB8 3PH, UK

**Keywords:** probiotics, biofeedback, electroencephalography, cognitive test, football players

## Abstract

Competitive football players who undergo strenuous training and frequent competitions are more vulnerable to psychological disorders. Probiotics are capable of reducing these psychological disorders. The present study aimed to determine the effect of daily probiotics supplementation on anxiety induced physiological parameters among competitive football players. The randomized, double-blinded, placebo-controlled trial was conducted on 20 male footballers who received either probiotics (*Lactobacillus Casei* Shirota strain 3 × 10^10^ colony forming units (CFU) or a placebo drink over eight weeks. Portable biofeedback devices were used to measure the electroencephalography, heart rate, and electrodermal responses along with cognitive tests at the baseline, week 4, and week 8. Data were statistically analyzed using mixed factorial ANOVA and results revealed that there is no significant difference between the probiotic and placebo groups for heart rate (61.90 bpm ± 5.84 vs. 67.67 bpm ± 8.42, *p =* 0.09) and electrodermal responses (0.27 µS ± 0.19 vs. 0.41 µS ± 0.12, *p* = 0.07) after eight weeks. Similarly, brain waves showed no significant changes during the study period except for the theta wave and delta wave at week 4 (*p <* 0.05). The cognitive test reaction time (digit vigilance test) showed significant improvement in the probiotic group compared to the placebo (*p <* 0.05). In conclusion, these findings suggest that daily probiotics supplementation may have the potential to modulate the brain waves namely, theta (relaxation) and delta (attention) for better training, brain function, and psychological improvement to exercise. Further research is needed to elucidate the mechanism of current findings.

## 1. Introduction

The effect of probiotics on human health is evidenced by many types of research on humans and animals. Probiotics provide not only physiological benefits but also psychological benefits to the individuals. Recently, many studies revealed the effect of probiotics on psychological conditions such as stress, anxiety, irritable bowel syndrome (IBS), and depression [1,2,3]. This novel concept led to the formation of a new term called “Psychobiotics”. Psychobiotics are the probiotics that contain psychotropic properties. If the right amount is consumed, it can positively affect the brain by providing benefits to the people who suffer from chronic stress, bad mood, or anxiety-like symptoms [4]. Abdullah [5] reported that gastrointestinal discomforts such as abdominal bloating are common among most populations which can affect their psychological disturbances. The microbiota-gut-brain axis is one of the vastly researched areas in the recent decade. The bi-directional relationship between the gut and brain captured the attention of many researchers, and they started to explore the depth of this relationship. Hence, any changes that occurred to the brain can affect the gut and vice versa [3,6]. However, there are still many areas that remained undiscovered.

The human gut is colonized by more than 100 trillion microbiotas, and these bacteria can provide benefits to the individuals, but they can also cause harm [7]. Probiotics are known as friendly bacteria living in the gut, and through correct administration, these probiotic bacteria can provide various health benefits to the host [8,9]. A human possesses a unique probiotic profile in the gut from birth, but probiotics can be consumed externally via fermented food, capsules, drinks, shots, and powder. Probiotic cultures that can tolerate the processing conditions of the digestion and show probiotic properties after the process with a maximum surviving capacity are considered as effective probiotics and *Lactobacillus Casei* Shirota strain is considered as one of the effective probiotic strains [10].

According to Huang et al. [9], the gut can manipulate the brain in different ways. Probiotics secrete neurotransmitters such as gamma-aminobutyric acid (GABA), serotonin, and catecholamines, which inhibit the transmissions of the nerve impulses in the central nervous system (CNS). Nevertheless, probiotics can regulate the dysfunctional hypothalamic–pituitary–adrenal axis (HPA axis). When an individual experience a stressful condition, the HPA axis activates and causes the adrenal glands to secrete cortisol. In inflammation, transmitters send impulses to the brain through the Vagus nerve causing psychological disorders, but probiotics are capable of minimizing the inflammation condition by improving the immune functions [3,10,11].

The psychophysiological studies associated with sports performance has been receiving increasing attention in the last 20 years [12]. Studying psychophysiology is a multidisciplinary approach, which focuses on the relationship between an individual’s mind and body [13]. Psychological disorders such as stress, anxiety, and depression can trigger the autonomic nervous system (ANS), which activates the sympathetic nervous system causing changes in the peripheral skin temperature, heart rate, respiration rate, electrodermal activities, muscular activities, electroencephalography (EEG) [14], and hormonal reactions such as cortisol level [15]. Psychological disorders can be measured using physiological methods, psychological methods, and biochemical methods, as it can affect the autonomic nervous system and endocrine system by activating the hypothalamic-pituitary-adrenal axis [16,17]. Physiological components can be measured objectively, through central measures via electroencephalography (EEG) and peripheral measures via electromyography (EMG), electrocardiography (ECG), electrodermal responses (EDR), peripheral skin temperature, and respiration rate [13], which are non-invasive and easily used in the lab and field settings.

Heart rate (HR) can be affected by stress and anxiety. Heart rate is controlled by the sympathetic nervous system whereby, when individuals are more aroused, the heart rate goes up and when relaxed, it comes down. The normal heart rate of an individual can be around 70 bpm [18], but athletes tend to have lower resting heart rate levels, and, in some cases, it can be around 40 bpm. Due to the advanced and easy to use mobility devices, HR can be measured in real-time in the playing field. Electrodermal response (EDR) or Galvanic skin response (GSR) is the measurement of the electrical conductance of the skin due to the sweat gland activities. When individuals encounter stress, the sympathetic nervous system gets activated, and it causes skin conductivity to increase.

Electroencephalography (EEG) can provide greater information about brain wave activities such as determining the dominant wave during different situations and from which part of the brain (frontal, temporal, parietal, and occipital). A study conducted by Baumeister, Reinecke, Liesen, and Weiss [19], reported that elite golfers show their highest performance with the increment of the upper alpha band in the partial part of the brain and theta in the frontal. Even though many studies focused on alpha brain waves, Park et al. [20] suggested that it is necessary to analyze all the brain waves namely alpha, beta, gamma, theta, and delta, simultaneously and their interactions to interpret the brain oscillation data better.

Gamma brain wave (above 30 Hz) is the fastest band, which is of high frequency, associated with the cognitive, attention tasks, and processes information from different brain areas. Beta brain wave (15–30 Hz) is the next fast band in line and is associated with consciousness, active thinking, attention, focus, and anxiousness. Beta is high when individuals engage in problem-solving and critical thinking. Alpha brain wave (8–12 Hz) indicates the awareness and relax state of the brain; these waves aid the calm and relax phase. According to Park et al. [20], alpha brain wave is the most dominant brain wave in adults and the alpha band is extensively studied in the literature. Theta brain wave (4–8 Hz) can be detected in deep relaxation, sleep, and meditation. Delta wave (0–4 Hz) is the slowest and low-frequency band associated with deep sleep and dreamless sleep [21,22].

Most of the EEG related studies in sports are associated with sports that need more psychological power than physical exertion such as precision measuring sports such as golf, dart throwing, archery, rifle shooting, and billiard [13]. Differences have been detected in the EEG absolute power due to the changes in the stress level, and EEG can accurately measure an individual’s stress [23]. Cooke et al. [24] found that expert golfers possess low EEG alpha waves and HR compared to novices. However, when they show low alpha waves, their performance accuracy is usually high. Even though many physiological responses due to stress and anxiety can be measured, the current study only focused on three anxiety-induced physiological measures, which are the brain wave responses, heart rate, and electrodermal responses.

Every sport comprises unique physical, psychological, and technical requirements and football is a team sport where players are required to produce maximal or sub-maximal effort for a prolonged period [25]. A study conducted by Junge and Feddermann-Demont [26] revealed that the prevalence of depression and anxiety symptoms are high among footballers. Thus, it is necessary to prevent psychological conditions among athletes to help them achieve their peak performance during competitions [27]. According to Huang et al. [9], there are many ways to treat anxiety disorders, for instance, using medications (antidepressants, beta-blockers, and herbs), counseling, and behavioral therapies. All these treatments have advantages and disadvantages. Hence, it is important to seek something that does not have side effects, easy to use, and low in cost and delivers a good outcome.

Consuming food is one of the ways to avoid the side-effects of medication, cost-effective, and it is also easily accessible [28]. However, the food-drug interaction needs to be carefully addressed [29], and the commercially available food must be properly regulated [30]. Commercially available probiotics are considered as a safe supplement for the athletes, and these can be used as nutritional therapy to treat psychological disorders [31]. Most of the other practiced methods such as psychological and biochemical methods to alleviate psychological conditions are associated with severe side effects, high cost, and some are time-consuming while probiotics are claimed as being easy to use, commercially available, and they are low-cost supplements with several other physiological benefits. Many recent studies have found the effectiveness of probiotics to manage stress, anxiety, and depression among different study samples [32,33,34,35,36,37,38,39,40,41].

Moreover, a study conducted by Kelly [42] showed that there is no significant effect of eight weeks of probiotic supplementation on EEG responses among healthy individuals. However, Wang [43] reported that theta and alpha oscillations were significantly high in the probiotic group after four weeks of supplementation among healthy volunteers. In a similar approach, Allen [44] reported that the theta brain wave was significantly low after four weeks of probiotic supplementation. Furthermore, probiotic supplementation significantly increased delta oscillation and improved sleeping in medical students [45]. It was evident that probiotics affect brain wave responses in different ways; thus, need more studies to conclude the outcomes. Due to the limited number of studies conducted so far to identify the effect of probiotics on athletes’ psychophysiological parameters, the current study is designed to identify the effect of eight weeks of probiotic supplementation on selected psychophysiological parameters among competitive football players.

## 2. Materials and Methods

### 2.1. Research Design

A randomized, double-blind, placebo-controlled study was conducted. Participants were randomly divided into two research conditions where the probiotic group daily received a probiotic supplement and the placebo group received a placebo supplement over eight weeks. The randomization was done using a computerized randomization software (www.randomizer.org) to randomly divide them into two research conditions. Selected psycho-physiological parameters were collected three times during the study simultaneously with the body composition, anthropometric measure, and diet records. Ethical approval was received from the human research ethics committee of Universiti Teknologi MARA, Shah Alam, Malaysia (600-IRMI (5/1/6)) to conduct the present study. Furthermore, the study was conducted following the guidelines of the International Declaration of Helsinki.

### 2.2. Participants

Twenty (*n* = 20) right-handed young adult male football players aged 18 to 21 were voluntarily selected for the study after being screened through an inclusion and exclusion criteria. Football players who had more than five years of football playing experience and were physically and mentally sound were included in the study while excluding the football players who had a history of psychological disorders, cardiovascular diseases, respiratory diseases, and gastrointestinal diseases. Furthermore, football players with known intolerance to probiotics and milk products were also excluded from the study as the treatment was a probiotic supplement. Smokers and antibiotic users nearly before the study begins were also excluded from the study. The participants voluntarily participated in the study after signing the consent form. The sample was recruited from one team (Universiti Teknologi MARA Football Club-UiTM FC) to avoid competition and training affects and all the selected participants were from the same ethnic and religious group. Due to that, food practices are similar among the participants. A similar approach was taken by the previous researchers to select the study samples similar to the present study [39,44,46].

The sample size was estimated using the G*Power 3.0.10 software and to achieve an 80% statistical power with a sample effect size of 0.35, with the significant level of 0.05, the proposed study required eight participants per group and assuming the 20% dropout rate, 10 participants were recruited for each group, with the total sample size of 20 participants. The sample size calculation was performed referring to the studies conducted by Allen et al. [44], Kelly et al. [42], and Sashihara et al. [47].

### 2.3. Intervention

*Lactobacillus Casei* Shirota strain was used as the probiotic supplement in the present study. The probiotic group (*n* = 10) daily received commercially available probiotic cultured milk (80 mL/bottle) with 3 × 10^10^ CFU live cells of *Lactobacillus Casei* Shirota strain mixed with commercially available orange fruit juice (120 mL) and the placebo group daily received a supplement that only contained commercially available orange fruit juice (200 mL). Both probiotics and orange drinks are commercially available and approved beverages by the Ministry of Health, Malaysia. Both groups received the same amount of supplement (200 mL) where the color and smell of the drinks are similar which supports the double-blinded process.

*Lactobacillus Casei* Shirota strain (LcS) was generally recognized as a safe probiotic strain and has been approved by the Food and Drug Administration of the USA [32]. A study conducted by Sheehan [48] revealed that *Lactobacillus Casei* could survive more than eight weeks after mixing with orange juice with a good number of bacteria in it such as 10^7^ CFU. Furthermore, orange juice can mask the perceptible off-flavor that is given by the probiotics while consuming at longer periods [49]. Hence, orange juice was used as the placebo drink and to mix with the probiotic drink for the intervention. To maintain the blinding process, the supplements were supplied by the researchers but distributed by an independent person, who is not involved in the research.

### 2.4. Instruments

Portable electrophysiological devices were used to gather anxiety-induced physiological parameters in the present study along with a cognitive test.

#### 2.4.1. Electroencephalography

The Muse EEG headband from InteraXon Inc, Toronto, ON, Canada, which was developed in 2003 and released in 2014, was used to acquire electrical activities of the brain in the present study. This is a non-invasive, portable, and easy to use EEG measuring device, which is validated for the research by Krigolson, Williams, Norton, Hassall, and Colino [50] and used by many researchers [22,51,52,53,54].

The Muse EEG headband has seven electroencephalography sensors where four sensors act as channels and the other three sensors as references. The Muse headband should be worn above the earlobes and three reference sensors should be located in the middle of the forehead. The band can be adjusted according to an individuals’ head size. Two sensors are located in the forehead, anterior frontal-7 (AF 7) in the left forehead and anterior frontal-8 (AF 8) in the right forehead, and the other two sensors that are above the ear lobe temporal-parietal-9 (TP 9) is above the left ear and temporal-parietal-10 (TP 10) above the right ear. These four sensors provide the electrical activities of the brain (alpha, beta, gamma, theta, and delta) and the Fpz or the reference sensor provides information about the accelerometer, blinks of the eye, movements of the jaw, and head. The device has a rechargeable lithium-ion battery and data were transmitted to the mobile app via Bluetooth technology.

The individual was instructed to sit comfortably in a chair and was given a few minutes to relax in the position, and then the EEG headband was placed on an individual’s head and adjusted accordingly. Participants were instructed to minimize the movements and eye blinks and keep the head still to avoid artefacts as much as possible. The Mobile Muse App indicates the signal from all the four sensors and recording was only started after making sure of the accurate signal from all the sensors. The EEG recording was done five minutes simultaneously with the cognitive task. Data were cleaned and artefacts removed before further analyzing. The absolute mean power for each frequency band was calculated by taking the average from four sensors (Equation (1)).

(1) $$\mathrm{Absolute}\;\mathrm{Alpha}\;\mathrm{power}\;=\frac{\mathrm{Alpha}\;\mathrm{AF}7+\;\mathrm{Alpha}\;\mathrm{AF}8+\;\mathrm{Alpha}\;\mathrm{TP}9+\;\mathrm{Alpha}\;\mathrm{TP}10}{4}$$

#### 2.4.2. Electrodermal Responses

The electrical conductance of the skin due to the sweat gland activities was measured using the NeuLog™ galvanic skin response logger sensor-NUL 217. Two electrodes with Velcro finger connectors are directly connected to the galvanic skin response (GSR) sensor. The GSR module was then connected to the USB-200 modules, which is connected to the PC via a USB cable. NeuLog (NUL 217) software was installed in the PC and all the connections and data accuracy were checked before use in the study. NeuLog is widely used by researchers to get physiological responses in sports and clinical settings [55]. Individuals were instructed to sit comfortably and two electrodes with Velcro connectors were placed tightly on the middle phalanx of the index finger and ring finger in the non-dominant hand. Data were given in Micro Siemens (µS) and the recording was conducted around five minutes simultaneously with the cognitive task where data were saved in a comma-separated values (CSV)file.

#### 2.4.3. Heart Rate

Heart Rate (HR) is directly associated with psychological disorders and measured as beats per minute (bpm). In the present study, the NeuLog™ Heart Rate sensor was used to measure the participants’ heart rate. Similar to the GSR module, the HR module has a sensor that can be attached to the little finger of the non-dominate hand. After the participant sits and relaxes in the position, the heart rate sensor is placed on the little finger and using the same software as the GSR, the heart rate was recorded five minutes simultaneously with the cognitive task. The present study procedure was similar to the research conducted by Plested, Gedeon, Zhu, Dhall, and Geocke [53].

#### 2.4.4. Cognitive Task

The digit vigilance test (DVT) was used as the cognitive task and as a stressor in the present study. DVT is a simple task that measures an individual’s sustained attention, vigilance, visual-motor tracking of an accurate selection of target stimuli. The DVT is a part of a larger battery of attention/psychomotor tests. Kelland and Lewis (1996), developed the DVT and proved its reliability and validity and indicated that DVT has good psychometric properties [56]. DVT is a paper and pencil test. The individual only needs to pay attention to the simple instruction given by the researcher. The test sheet contains 59 rows of 35 digits in each row, where 0 to 9 digits were randomly arranged in a 12-point (pt) font size in two A4 papers. The first task is to mark all the sixes in the first paper and the second task is to mark all the nines in the second paper; it generally takes 10 min to complete both tasks. In the present study, individuals were asked only to complete the first part of the task which only took 5 min or less to complete and the physiological measure (EEG, EDR, and HR) was recorded simultaneously. The correct number of hits (DVT accuracy percentage) along with the time (DVT reaction time) they spend to complete the task was calculated. More correct hits with less reaction time indicate the high sustained attention. DVT has been a popular cognitive test and it has been used by various researchers to measure sustained attention [57,58,59].

#### 2.4.5. Anthropometric Measures

Bodyweight was measured to the nearest 0.1 kg with a digital weighing scale (SECA model 803, Hamburg, Germany). Height was measured using a portable stadiometer (SECA model 213, Hamburg, Germany) to the nearest 0.1 cm. Body mass index (BMI) was calculated as kg/m^2^ only to see the uniformity between two research conditions at the baseline. This is because BMI can often misinterpret the body fat measurement, especially for athletes [60,61,62,63].

#### 2.4.6. Diet and Energy Intake

The three-day dietary record (3DDR) was used to analyze the diet and energy intake of the participants where individuals were asked to record their regular food intake during three consecutive days including two weekdays and one weekend day. The portion sizes of the foods consumed were estimated using household measurements. The Nutritionist Pro™ (Axxya Systems, Woodinville, WA, USA) software version 2.4.1 (First Data Bank INC., 2011) was used to analyze the diet based on the Malaysian food database. The Nutritionist Pro™ is a widely used software to analyze the energy and nutrient intake all over the world [64,65,66].

### 2.5. Study Procedure

Participants were thoroughly informed about the study procedures and were allowed to continue their regular food practices along with regular training and competitions. However, they were not allowed to take any probiotic products and antibiotics during the study. The supplement drinks were prepared each day one hour before distribution and kept cold at 4 °C. The football team trained daily at the UiTM football field throughout the study period, hence that provide a facilitative environment to provide and administrate the supplements. Every week they had a practice match and a gym session. Daily administration was done, and records were taken. According to Coqueiro et al. [66], daily probiotic supplementation is crucial, if an interruption occurs such as discontinuation for more than eight days the probiotics may no longer be detected in the gut. The treatment was continued for eight weeks and data were gathered at the baseline (week 0), fourth week (week 4), and eighth week (week 8).

Data were collected in a closed laboratory with room temperature at 25 °C. Participants were instructed to have a good night’s sleep and to avoid high intensity training the day before data collection. They were also instructed not to consume any caffeine products on the same day. Physiological data EEG, EDA, and HR were recorded simultaneously with the DVT cognitive task. The participants were instructed to sit comfortably with the non-dominant hand resting on the table, then NeuLog GSR sensor electrodes were placed on the middle phalanx of the index and ring finger and a NeuLog HR sensor electrode was placed on the little finger of the non-dominant hand. Next, the Muse EEG headband was placed on the head accurately and adjusted according to the head size. The researcher checked for all the connections and made sure that the signals were clear. The participants were asked to minimize the movements and keep the head still as much as possible while recording data. Then, the DVT task was introduced and simple instructions were given verbally even though it was written on the paper. Five minutes were given to relax in the position and data were recorded when the participant started the DVT task. At the end of the DVT task, data recording was stopped and saved in a CSV file format. This same procedure was done at the baseline and after the fourth and eighth week of the experiment in the same place with the same conditions.

### 2.6. Data Analysis

Data were analyzed using the SPSS (version 25.0) statistical software. According to the Kolmogorov-Smirnov test, data were normally distributed due to the parametric tests that were used. The mixed factorial analysis of variance (mixed between within ANOVA) test was conducted with time (week 0, week 4, week 8) as the within-subject factor and group (probiotic vs. placebo) as the between-subject factor. Bonferroni correction was used to measure the differences between time points over groups (post-hoc analysis). Furthermore, the independent sample *t*-test was conducted to analyze the between group differences at the baseline. The significance *p*-level was set at 0.05.

## 3. Results

Out of 20 participants, one player from the placebo group withdrew from the study and the reason was not associated with the research. None of the participants had reported using antibiotics, side effects, or adverse conditions during the probiotic supplementation. In Table 1, no significant changes were detected at the baseline.

The mixed factorial ANOVA test was conducted with time as the within-subject factor and group as the between-subject factor. According to the results, statistically significant changes were detected for the within groups factor for beta brain wave, gamma brain wave, and delta brain wave and results are presented in Table 2. None of the other brain waves showed significant interactions for time or time*group factors.

According to the mixed factorial ANOVA, heart rate and electrodermal responses showed statistically significant changes over time. Furthermore, the DVT reaction time significantly changed over time but no changes were detected for the time*group interaction. Results are presented in Table 2.

Results revealed that there are significant differences within groups over time for most dependent variables. Hence, a pairwise comparison was conducted with Bonferroni adjustment and both main effects and simple effects were reported in the present study. Results were presented in Table 3.

### 3.1. Effect of Probiotics on Brain Wave Activities

**Figure 1:**
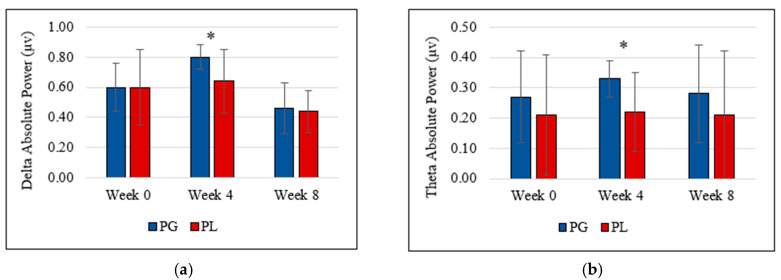
(**a**) Delta absolute power and (**b**) theta absolute power deviation during the intervention. (PG = Probiotic Group, PL = Placebo Group).

Data showed no gradual increment or decrement and no statistically significant difference between probiotic and placebo groups at the end of the study (week 8). However, the probiotic group showed a significant increment of theta brain wave (0.33 µV ± 0.06 vs. 0.22 µV ± 0.13, *t*(17) = 2.391, *p* = 0.029, partial eta^2^ = 0.25) and delta brain wave (0.80 µV ± 0.08 vs. 0.64 µV ± 0.21, *t*(17) = 2.102, *p* = 0.043, partial Eta^2^ = 0.22) at week 4 compared to the placebo group. Figure 1a,b represents the delta and theta power following the probiotics supplementation.

### 3.2. Effect of Probiotics on Electrodermal Responses and Heart Rate

**Figure 2:**
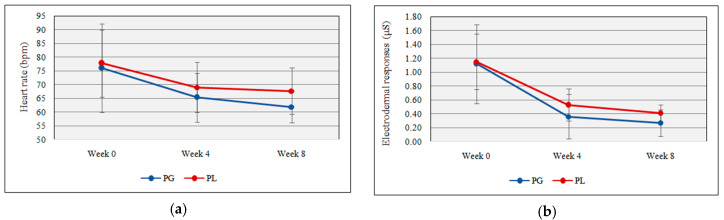
(**a**) Heart rate response deviation throughout the study; (**b**) electrodermal response deviation throughout the study (PG = Probiotic Group, PL = Placebo Group).

NeuLog™ GSR and HR sensors provide readings for electrodermal activities and heart rate. No significant difference was shown between the probiotic and placebo group for electrodermal responses (0.27 µS ± 0.19 vs. 0.41 µS ± 0.12, *t*(17) = −1.89, *p* = 0. 07) and heart rate responses (61.90 bpm ± 5.84 vs. 67.67 bpm ± 8.42, *t*(17) = −1.75, *p* = 0.09) with the daily probiotic supplementation. Figure 2a,b shows the heart rate and electrodermal responses following probiotics supplementation.

### 3.3. Cognitive Task

**Figure 3:**
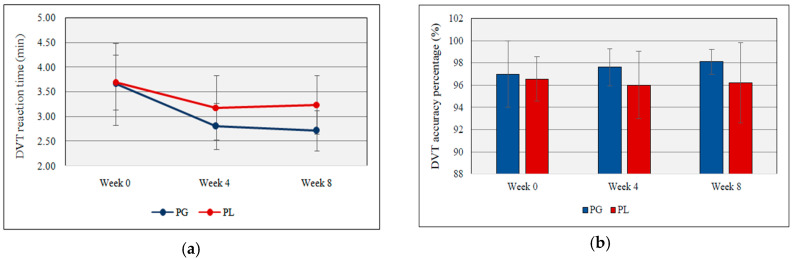
(**a**) DVT reaction time during the study period; (**b**) DVT accuracy percentage during the study period (PG = Probiotic Group, PL = Placebo Group).

Figure 3a,b illustrates the reaction time and accuracy percentage of DVT. Both groups gradually decrease the DVT reaction time but at week 8, there was a significant difference between probiotic and placebo groups (2.71 min ± 0.41 vs. 3.24 min ± 0.59, *t*(17) = −2.26, *p* = 0.037, partial Eta^2^ = 0.23). The accuracy percentage of the DVT was calculated by counting the number of correct hits and missed hits. No significant changes were detected between probiotic and placebo groups during the supplementation.

### 3.4. Anthropometric Measures and Diet Intake

Anthropometric measures, energy intake, and macronutrients showed no significant difference between two research groups at the baseline and the end of the study.

## 4. Discussion

Brain waves respond differently to different stimuli, and individuals have unique patterns. Even though no significant differences were found between the two groups during the study period, absolute theta power and delta power were found to be significantly high in the probiotic group compared to the placebo group at the fourth week of supplementation. The theta oscillation is one of the slowest brain waves where the frequency was around 4–8 Hz that could detect when an individual felt more relaxed, in a sleeping state or while meditating. A study conducted by Amirtham and Saraladevi [67] recruited eight ADHD (attention deficit and hyperactive disorder) children to analyze the effect of relaxation therapy on brain waves and revealed that theta wave was significantly high after the relaxation treatment. Similarly, Jacobs and Friedman [68] reported that relaxation techniques lowered the arousal level while significantly increased the theta brain wave among the experiment group proving the correlation between these two components. Nevertheless, meditation is another method that decreases stress and anxiety while increasing relaxation. Aftanas and Golocheikine [69] found that meditation increases theta frequency which again shows the positive correlation between theta brain wave and relaxation. Nevertheless, a review by Schacter [70] explained the relationship between the high theta activity and psychological processes, which stressed the negative correlation between stress and theta oscillation. The present study results showed that absolute theta oscillation was significantly high among the football players in the probiotic group compared to the placebo group at week 4, similar to the study conducted by Wang [43]. This indicated that the football players in the probiotic group were more relaxed with low stress and anxiety.

In the present study, delta oscillation is significantly high in the probiotic group compared to the placebo at week 4. High delta responses were often associated with the cognitive process such as attention, problem solving, perception, and signal tracking [71]. In contrast, absolute alpha, absolute beta, and absolute gamma powers were low in the probiotic group compared to their counterpart. Thus, these changes were considered as non-significant. Knyazev in 2007 reported that increased theta power and decreased alpha and beta power were associated with relaxation and also highlighted that low alpha levels can be detected in high attention situations [72].

There are a limited number of studies that had been conducted to identify the effect of probiotics supplementation and EEG responses. In a similar study approach to the present study, Kelly et al. [42] reported that 29 healthy male participants underwent a randomized placebo-controlled cross-over probiotic (*Lactobacillus Rhamnosus*, 1 × 10^9^ CFU) supplementation intervention and there were no significant differences observed on any electroencephalogram (EEG) parameters after four weeks of probiotic supplementation, but in the present study there were significant differences for theta and delta waves in the probiotics supplemented group after week 4. In another study conducted by Allen et al. [44], the measured psychological, electrophysiological, and cognitive responses with probiotic supplementation (*Bifidobacterium longum* 1714 strain 1 × 10^9^ CFU) had reported a significant decrease in the theta power after being supplemented with probiotics for four weeks. The findings were not comparable with the present study as the theta power was significantly high in the probiotic group. This may be due to the usage of different probiotic strains, research design, and devices in two studies. Allen et al. [44] also conducted cognitive tests along with EEG recording and paired-associate learning tasks, which was conducted to measure conditional learning, and it was revealed that errors were lowered in the probiotic period compared to the placebo.

Zafar et al. [73] conducted a study to identify the responses of the EEG frequency band on cognitive tasks and reported that the delta frequency band increased with attention. Many researchers reported that an increased delta brain wave correlates with decision making and attention [71,72,73,74]. All these findings showed a better-sustained attention by significantly improving the DVT reaction time that concurs with present study findings.

In alpha power, the present study did not have any reduction following probiotics supplementation. Amirtham and Saraladevi [67] and Aftanas and Golocheikine [69] reported that relaxation therapy and meditation, respectively have significantly increased alpha absolute power that indicates an improvement in relaxation. While a study conducted by Jacobs and Friedman [68] reported that theta brain wave was significantly higher with relaxation techniques whereas the alpha band did not show a significant decrement in different brain regions such as frontal, partial, occipital, central, and temporal during relaxation. Hence, theta can be a good predictor of relaxation compared to the alpha frequency band. Similarly, Kubota et al. [75] detected a high theta power and non-significant low alpha power in 25 participants who underwent a relaxation experiment.

Domingues et al. [76] reported that absolute alpha power was inversely correlated with attention. The study was conducted using 23 male shooters, whose EEG were recorded while the participants performed pistol shooting. This phenomenon was supported by many studies [77,78,79]. Due to that, it can be deduced that lower alpha power indicates better attention according to these studies. Most studies focused on targeting sports such as archery, shooting, golf, and dart throwing when analyzing brain waves and cognitive functions. Thus, there was a considerable gap in identifying brain wave functions in football and other sports. In the present study, football players in the probiotic treatment group showed low anxiety and stress with a better reaction time for sustained attention. These psychological and cognitive components can be related to brain wave patterns that were shown during the experiment period.

Both the probiotics and placebo groups showed a significant decrement in electrodermal responses and heart rate between week 0 and week 4. Heart rate and electrodermal responses were recognized as good physiological feedbacks to stress [80]. Athletes possess lower heart rates compared to sedentary people, and according to Baggish and Wood [81], HR can be in the range from 40 to 200 bpm. In the present study, football players showed lower heart rates than the average people.

Heart rate and electrodermal responses showed a gradual decrement in both groups during the study period, however, no significant difference was noted between the two groups at the three data collection points (week 0, week 4, and week 8). In the present study, even though the researchers were well acquainted with the research instruments among football players before using them in data collection, it could be possible that the football players felt nervous during the first data collection. That may be the reason for the rapid fall of heart rate and electrodermal responses between week 0 and week 4 data collections. Civitello et al. [82] stated that when the experiment started, the participants showed nervousness over the instruments that were used to obtain physiological feedbacks, and it may affect the baseline data. A study reported that heart rate and electrodermal responses were high in 31 university students in stressful conditions [82]. A study conducted by Sharma, Goel, Srivastav, and Dhasmana [83] showed that heart rate and electrodermal responses were high in critical care unit nurses compared to others. This indicates the relationship between stress and physiological feedbacks. These studies indicate the physiological responses over psychological stimuli. Thus, the current results showed no significant changes in EDR and HR with daily probiotic supplements.

In the present study, the daily probiotic supplementation was able to increase the vigilance of the football players. The individuals with anxiety and depression tend to show slower reaction times due to the dysfunction of the process to communicate accurate information accompanied by anxiety and depression [84]. According to the results, even though the probiotic group showed gradual improvement in the DVT accuracy percentage during the study, the difference between the two experimental groups was not significant. Kelly et al. [42] investigated the effect of probiotics on stress by analyzing the EEG, cognitive processes, and biomarkers. They recorded EEG while the participants engaged in cognitive tasks (paired associate learning, attention switching task, rapid visual information processing, emotional troop, and emotional recognition task). Thus, the cognitive outcomes from Kelly et al.’s study showed no significant difference in the probiotic supplement group, which were contradictory findings to the present study.

Chung et al. [85] in a placebo-controlled study involving 47 healthy older adults, administered a daily probiotic (*Lactobacillus helveticus*) supplementation over 12 weeks. The results revealed that the digit span test (DST), which was used to measure attention, showed no significant difference between the probiotic group and placebo. Rijken et al. [86] reported that using the device such as biofeedback (training to maintain heart rate) and neurofeedback (training to control brain waves) had improved the performance of the football players while reducing stress and anxiety. Similarly, Rusciano, Corradini, and Stoianov [87] identified the importance of regulating the psychophysiological components to overcome injuries in football. All these efforts were taken to reduce psychological disorders using physiological methods. Probiotics seem to have psychometric properties to regulate psychophysiological disorders while improving cognitive levels among individuals [88].

## 5. Conclusions

Football is a game where players need to make decisions based on a rapidly changing environment while considering teammates, opponents, as well as the ball. Due to that, players need to intensely focus their attention on the game and maintain the highest physical capabilities to achieve their targets. Stress, anxiety, and depression are often associated with competitions, and regulating these psychophysiological components can be the solution to improve performance via food-based nutritional supplements. Though the heart rate and electrodermal responses showed no significant changes with daily supplementation of *Lactobacillus Casei* Shirota strain, the delta and theta brain waves were higher in the probiotic group after four weeks that provide evidence for relaxation and attention components in the probiotic group. Sustained attention also showed significant improvement in the probiotic group with supplementation. As far as we know, this is the first report to clearly show the beneficial effects of probiotics supplementation on brain wave outcomes using objective EEG measures. The complete mechanisms of action of probiotics supplementation remain unknown. Further studies are needed to clarify the mechanisms underlying the effects of probiotics supplementation on maintaining psychology outcomes via brain waves.

## Figures and Tables

**Table 1:** Physical characteristics measurements of the football players at the baseline.

Parameter	Probiotic Group (*n* = 10)	Placebo Group (*n* = 9)
Age (years)	19 ± 0.81	19 ± 0.66
Training (years)	7.9 ± 2.99	7.5 ± 2.67
Height (cm)	169.86 ± 5.74	169.05 ± 4.87
Weight (kg)	62.03 ± 5.31	65.82 ± 7.22
BMI (kg/m^2^)	21.59 ± 2.45	22.58 ± 2.20

Mean ± SD.

**Table 2:** Between and within group interaction of the study variables.

	Time				Time*Group			
	Wilks’ Lambda	*F*	*p-* Value	Partial Eta^2^	Wilks’ Lambda	*F*	*p-* Value	Partial Eta^2^
Delta	0.51	7.67	0.00 *	0.48	0.83	1.62	0.22	0.16
Theta	0.93	0.52	0.60	0.06	0.96	0.27	0.76	0.03
Alpha	0.75	2.59	0.10	0.24	0.97	0.18	0.83	0.02
Beta	0.54	6.79	0.00 *	0.45	0.97	0.23	0.79	0.02
Gamma	0.43	10.52	0.00 *	0.56	0.90	0.81	0.46	0.09
HR	0.53	7.10	0.00 *	0.47	0.97	0.24	0.78	0.03
EDR	0.25	23.39	0.00 *	0.74	0.97	0.18	0.83	0.02
DVT RT	0.42	10.81	0.00 *	0.57	0.88	1.05	0.37	0.11
DVT AP	0.98	0.12	0.88	0.01	0.91	0.76	0.48	0.08

Significant at *p* < 0.05 *; HR: Heart rate; EDR: Electrodermal responses; DVT RT: Digit vigilance test reaction time; DVT AP: Digit vigilance test accuracy percentage.

**Table 3:** Pairwise comparison for the between group effect.

	Week 0		*p*	Week 4		*p*	Week 8		*p*
	Probiotic	Placebo		Probiotic	Placebo		Probiotic	Placebo	
Alpha	0.38 ± 0.12	0.45 ± 0.14	0.32	0.35 ± 0.08	0.38 ± 0.06	0.40	0.41 ± 0.13	0.47 ± 0.14	0.33
Beta	0.29 ± 0.18	0.34 ± 0.20	0.62	0.21 ± 0.11	0.29 ± 0.16	0.26	0.30 ± 0.11	0.41 ± 0.19	0.14
Gamma	0.04 ± 0.27	0.04 ± 0.21	0.98	−0.11 ± 0.18	−0.01 ± 0.19	0.27	0.09 ± 0.15	0.12 ± 0.24	0.69
Theta	0.27 ± 0.15	0.21 ± 0.20	0.50	0.33 ± 0.06	0.22 ± 0.13	0.02 *	0.28 ± 0.16	0.21 ± 0.21	0.43
Delta	0.60 ± 0.16	0.60 ± 0.25	0.95	0.80 ± 0.08	0.64 ± 0.21	0.04 *	0.46 ± 0.17	0.44 ± 0.25	0.80
EDR	1.12 ± 0.57	1.15 ± 0.40	0.93	0.36 ± 0.32	0.53 ± 0.23	0.20	0.27 ± 0.19	0.41 ± 0.12	0.07
HR	76.00 ± 16.14	77.78 ± 12.23	0.79	65.30 ± 8.93	69.00 ± 9.18	0.38	61.90 ± 5.84	67.67 ± 8.42	0.09
DVT RT	3.66 ± 0.83	3.69 ± 0.56	0.93	2.80 ± 0.47	3.18 ± 0.65	0.16	2.71 ± 0.41	3.24 ± 0.59	0.03*
DVT AP	97.00 ± 2.98	96.55 ± 2.00	0.71	97.60 ± 1.65	96.00 ± 3.04	0.16	98.10 ± 1.10	96.22 ± 3.59	0.16

Significant at *p* < 0.05 *; HR: Heart rate; EDR: Electrodermal responses; DVT RT: Digit vigilance test reaction time; DVT AP; Digit vigilance test accuracy percentage.
